# Long-Term Spasticity Management in Post-Stroke Patients: Issues and Possible Actions—A Systematic Review with an Italian Expert Opinion

**DOI:** 10.3390/healthcare11060783

**Published:** 2023-03-07

**Authors:** Giovanni Morone, Alessio Baricich, Stefano Paolucci, Anna Rita Bentivoglio, Paolo De Blasiis, Matilde Carlucci, Francesco Violi, Gabriella Levato, Marcello Pani, Lucia Federica Carpagnano, Federico Spandonaro, Alessandro Picelli, Nicola Smania

**Affiliations:** 1 Department of Life, Health and Environmental Sciences, University of L’Aquila, 67100 L’Aquila, Italy; 2 San Raffaele Institute of Sulmona, 67039 Sulmona, Italy; 3 Physical Medicine and Rehabilitation, Department of Health Sciences, Università del Piemonte Orientale, 28100 Novara, Italy; 4 Santa Lucia Foundation, IRCCS, 00179 Rome, Italy; 5 Neuroscience Department, Policlinico Universitario Agostino Gemelli, IRCCS, 00168 Roma, Italy; 6 Department of Mental and Physical Health and Preventive Medicine, Section of Human Anatomy, University of Campania “Luigi Vanvitelli”, 80138 Naples, Italy; 7 Healthcare Directorate, AOUI Verona, 37126 Verona, Italy; 8 Internal Medicine Department, Sapienza Università di, 00185 Roma, Italy; 9 Health Protection Agency of Milan, 20122 Milan, Italy; 10 Policlinico Universitario Agostino Gemelli, IRCCS, 00168 Roma, Italy; 11 Hospital Medical Management Department, AOUI, 37126 Verona, Italy; 12 C.R.E.A. Sanità, University San Raffaele, 00166 Rome, Italy; 13 C.R.E.A. Sanità (Centre for Applied Economic Research in Healthcare), 00196 Rome, Italy; 14 Department of Neurosciences, Biomedicine and Movement Sciences, University of Verona, 37134 Verona, Italy; 15 Canadian Advances in Neuro-Orthopaedics for Spasticity Congress (CANOSC), Kingston, ON K7K 1Z6, Canada

**Keywords:** systematic literature review, PSS, rehabilitation care, BoNT-A, consensus, post-stroke spasticity management

## Abstract

Spasticity is a well-known motor dysfunction occurring after a stroke. A group of Italian physicians’ experts in treating post-stroke spasticity (PSS) reviewed the current scientific evidence concerning the state-of-the-art clinical management of PSS management and the appropriate use of botulinum toxin, aiming to identify issues, possible actions, and effective management of the patient affected by spasticity. The participants were clinicians specifically selected to cover the range of multidisciplinary clinical and research expertise needed to diagnose and manage PSS. When evidence was not available, the panel discussed and agreed on the best way to manage and treat PSS. To address the barriers identified, the panel provides a series of consensus recommendations. This systematic review provides a focused guide in the evaluation and management of patients with PSS and its complications. The recommendations reached by this panel of experts should be used by less-experienced doctors in real life and should be used as a guide on how to best use botulinum toxin injection in treating spasticity after a stroke.

## 1. Introduction

In Europe and worldwide, strokes are the second most common cause of death and a leading cause of long-term disability in adults [1].

An after-stroke rate survival is estimated to increase by 27% between 2017 and 2047 in the European Union [2]. The improvement in the survival rate leads to an increase in the prevalence of post-stroke complications and a greater need for specialized treatments.

Spasticity, in particular, is one of the main stroke complications. It is a motor disorder, a sub-class of the upper motor neuron syndrome, characterized by a rate-dependent increase in tonic stretch reflexes. Post-stroke spasticity (PSS) causes weakness, reduced motor control, pain, spasms, abnormal posture, and an overall decline in the subject’s quality of life [3]. PSS is observed in almost 25% of patients within 2 weeks after a stroke which increases to 38% after 12 months and to 44% in patients with recurrent hospitalizations [3,4,5,6].

A multidisciplinary approach combining physical rehabilitation with pharmacological interventions is required for successful PPS management [4]. Among pharmacological interventions, botulinum toxin type A (BoNT-A), the most potent neurotoxin known, is a safe and effective treatment for PSS in adult patients with stroke [7]. It has been clinically used for treating PSS in the last 30 years and is the accepted standard of care for focal post-stroke spasticity [8,9,10]. In recent analyses performed in Europe, a significant under-recording of PSS in primary care data, likely reflecting a poor diagnosis or signaling of this condition, has been reported [11]. As a matter of fact, unlike other common post-stroke complications, the UK Sentinel Stroke National Audit Programme (SSNAP) does not specifically monitor spasticity at any point in the stroke patient’s course [4]. Moreover, there is no nationally agreed pathway for PSS, and no stated protocols for the detection, monitoring, and referring of PSS patients or high-risk patients for this condition [4]. As a result, PSS patients are often only referred for spasticity treatment in case of secondary complications, reducing the possibility of benefiting from timely cost-effective treatments. Another problem limiting the management of post-stroke spasticity is the poor link between subacute hospital healthcare and community healthcare: the spasticity and related symptoms might typically regard subacute and chronic phases [12]. The absence of the “continuum of care” is one of the main problems of most healthcare systems as highlighted by a WHO initiative regarding the importance of the continuum of care for non-communicable diseases [13].

In light of this consideration, spasticity care represents a complex scenario for different disease phases and settings to pay attention to, for different medical and non-medical specialists involved, and for different treatments that should be organically managed. Previous research highlights some of these problems, focusing, for example, on the lack of awareness and knowledge of spasticity, insufficient access to spasticity services, and a lack of standardized processes/pathways [4]. Although other authors have emphasized the lack of timely or even early diagnosis, early detection and management of PSS, in fact, may not only reduce the disease impact but may help to prevent complications (i.e., joint contractures, decubitus, pain, and risk of falls) that can affect patients’ rehabilitation and quality of life [14,15]. Moreover, the lack of multidisciplinary care and the absence of a continuum of care, combined with the fact that there are few centers and specialists who treat spasticity, result in a patient’s withdrawal from spasticity treatment. Following this line, two recent Italian surveys of almost 80 clinicians specializing in spasticity show that the proportion of patients who fail to return for the second treatment with botulin toxin injection was reported to be 8.9–9.2%, and this was mainly due to unsatisfactory results, difficulty in reaching the hospital, and a lack of a standardized pathway [16]. In addition, the authors showed unmet clinical needs in terms of optimal timing for treatment, dosage selection, and suitable follow-up timing [16]. Italy, as well as other nations, does not have a consistent clinical care model for the treatment of post-stroke spasticity with BoNT-A and the routine use of botulinum toxin in clinics is far from standardized [17]. Notwithstanding a massive literature supporting the efficacy of BoNT-A for the treatment of PSS, there still are shortcomings in the routine management of PSS patients [18,19,20,21,22,23].

The aim of the present work is to improve the spasticity treatment pathway at an organizational and clinical level by means of mixed-method research based on a systematic review of the literature dealing with the issue of spasticity management and on the consent of a multidisciplinary experts’ panel.

## 2. Materials and Methods

**Figure 1:**
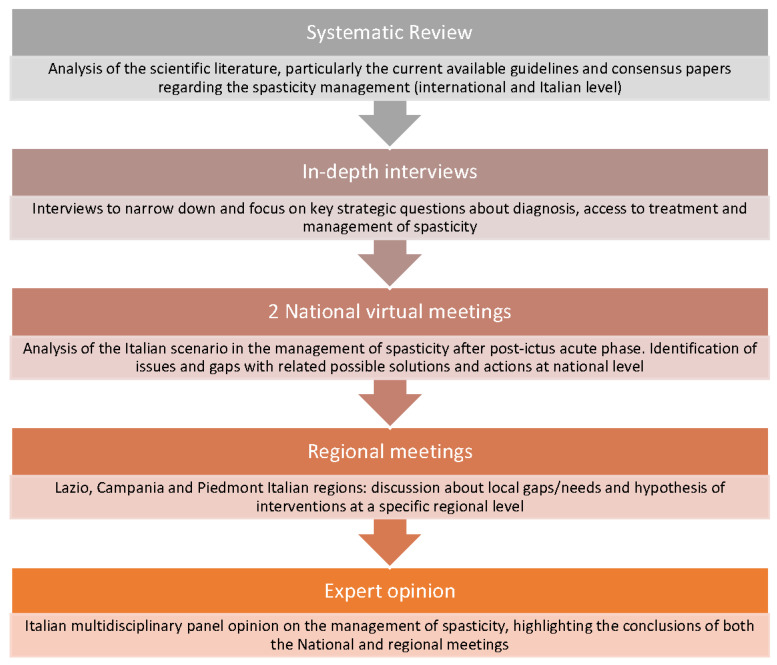
Mixed-method research flow: from the systematic review to identify key questions, to the expert opinion based on the national and regional multidisciplinary expert meetings.

This research is based on a mixed method, as already described in the literature [24,25], taking into account (1) a systematic review of the currently available international guidelines and consensus papers regarding spasticity management and (2) expert panels’ virtual meetings which involved a multidisciplinary panel of Italian physicians with expertise in the management of PSS. In order to take into account possible differences in the health system organization of the various Italian territories, physicians were chosen to be representative of four main Italy regions, namely in the south (Campania), center (Lazio), and north (north-west Piedmont, north-east Veneto) of Italy. Details on the mixed-method research flow are reported in Figure 1.

The consensus process is a collaborative and cooperative process where the group of experts is committed to finding the solution that best meets the opinion of the group.

The multidisciplinary panel includes physiatrists, neurologists, neurophysiopathologists, internists, GPs (general practitioners), health economists, and pharmacists, as well as regional health representatives. The meetings were intended to analyze the PSS settings, defining the primary intervention areas and the related solutions or recommendations.

This systematic review was conducted in line with the guidelines for reporting systematic reviews and meta-analyses (PRISMA) [26].

A systematic review of the existing literature and current guidelines on spasticity management was conducted, and the main review question was: “What are the possible solutions and recommendations to improve the management of spasticity in daily clinical practice?”

A systematic, comprehensive bibliographic search was carried out in the National Library of Medicine (Medline) and EMBASE databases for the period between 2000 and September 2022 in the PubMed database. The following keywords were mainly used: “post-stroke spasticity”, “post-stroke spasticity management”, “stroke guidelines”, “stroke management”, and “botulinum toxin”.

Inclusion criteria were as follows: papers published in a peer-reviewed journal, written in the English language, and published from 2000 to December 2022. Moreover, papers that report only human studies and that used a quantitative or qualitative methodology reporting spasticity management were included.

Studies that did not meet the inclusion criteria were excluded, while those that complied with the inclusion criteria were listed and further reviewed.

Studies were evaluated by two independent reviewers, GM and NS. Literature screening was performed by progressively reading the title, the abstract, and the full text. In cases of uncertainty, a discussion was held among the two authors that performed the screening to reach a common consensus.

The authors also considered guidelines about the management of PSS both published in peer-reviewed journals and/or in an official national website dedicated to the guidelines.

For each article, a data collection form was compiled which indicated: (i) author; (ii) title; (iii) type of study; (iv) purpose of the study; (v) brief description of materials and methods and results; (vi) conclusions; and (vii) level of evidence according to 2011 Oxford CEBM Levels of Evidence [27].

The focus of our medical systematic review is to provide an overview of all PSS management issues with the aim to improve real-life recommendations.

The most important world medical institutions were also directly evaluated for medical guidelines and a total of five guidelines were selected: the Canadian stroke best practice recommendations, the Royal College of Physicians, London, UK(UK); the American Heart Association (AHA), American Stroke Association (ASA); the New Zealand Guidelines Group and the SIGN Guidelines, Scotland. All the guidelines written in English that were selected are available in a complete format and are published in print or online.

An extensive and productive discussion of the national multidisciplinary expert panel analyzed the Italian scenario and identified organizational critical issues and gaps, confirming what had been already outlined by a set of preliminary in-depth interviews with key members of the expert panel.

The aggregated outputs of these interviews, made by an experienced consensus moderator not involved in the clinical practice of spasticity, were summarized and presented at the virtual meetings; controversies were solved by seeking an agreement between the experts.

The questionnaire focused on diagnostic issues, treatment access barriers post-diagnosis, and organizational aspects for optimal patient care management (Table 1). Related possible solutions and actions together with practical recommendations have been proposed.

The Regional Expert Panels confirmed the critical issues identified at the National level, thus suggesting interventions at a specific regional level.

## 3. Results

**Figure 2:**
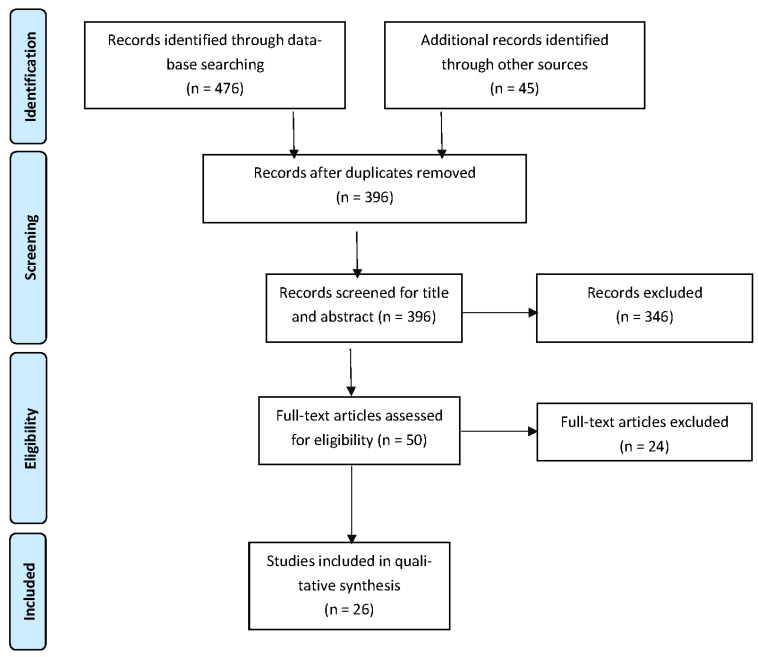
PRISMA 2020 flow diagram for the systematic review.

A total of 521 records were identified through database searching and other sources. A total of 26 articles (including 5 national guidelines) were selected, after duplicate removal and inclusion criteria evaluation, in this comprehensive systematic review (Figure 2).

The most relevant key messages of the selected studies on the topic of post-stroke spasticity management are reported (Table 2 and Table 3).

In particular, we have included five national guidelines [10,44,45,46,47], two systematic reviews [32,34], a randomized controlled trial [43], six observational studies [22,27,28,35,36,41], three non-systematic reviews [33,38,42], a comprehensive overview [29], consensus report [37], and three survey-based qualitative studies [31,39,40].

**Figure 3:**
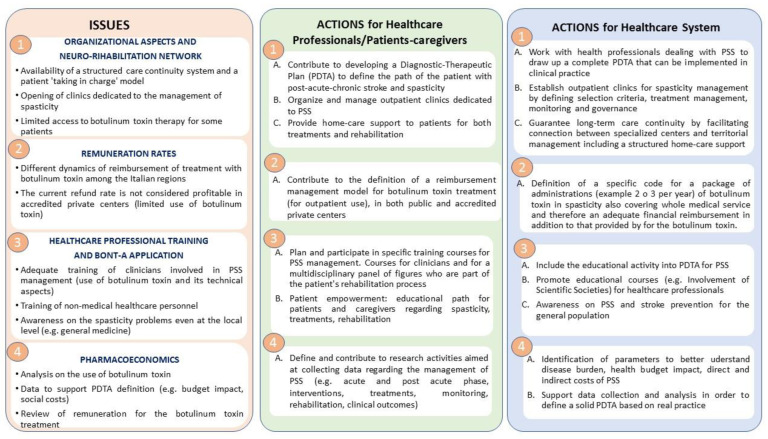
Overview of the results obtained with the Italian expert meetings. The figure shows issues identified (on the **left**); possible actions for healthcare professionals and for the patients/caregivers (in the **middle**); and possible actions for the healthcare system (on the **right**).

As to the results of the expert meetings, Figure 3 reports the most relevant issues identified by the panel. Furthermore, Figure 3 reports the actions needed in order to improve the current management of post-stroke spasticity.

## 4. Discussion

The aim of the present work is to improve the organizational and clinical aspects of spasticity treatment in real life, through a systematic review and a multidisciplinary expert opinion.

The panel of experts identified four main problems summarized in Figure 3: organizational aspects, differences in remuneration, healthcare professional training, and pharmaco-economic aspects. In terms of the organizational aspects, this raises an issue with respect to the overall management of stroke. In fact, most of the resources for stroke treatment are spent in the first temporal phase, the acute phase. On the other hand, the subacute and chronic phase is very poorly valued in terms of the allocated budget, which reflects assistance and organizational problems. For this reason, stroke should benefit from a pathway dedicated to pathology that does not stop only at the acute and subacute phases. In Italy, this pathway is identified by PDTA as a diagnostic therapeutic pathway. In addition, effective management of spasticity requires rigorous evaluation, patient-centered identification of goals, and additional physical treatments and should be followed by complementary physiotherapy treatment [33]. This complexity leads to the need for an organization of the territorial phase to be both inclusive of the different contexts where the patient is located (home, outpatient rehabilitation, chronic rehabilitation facilities, etc.) and at the same time specific and standardized for what concerns procedures and expertise (need for advanced diagnostic and therapeutic procedures such as EMG, phenol nerve block, post-injection casting, etc.). The panel of experts proposes the opening of territorial clinics dedicated to the multidisciplinary treatment of spasticity that is well integrated into the network and in the care flows of patients with both subacute and chronic stroke.

The matter of the limited number of patients with spasticity cared for also has a medical cultural basis: just think that there is no specific code for the diagnosis of spasticity (in the International Classification of Diseases, ICD-9 based on the World Health Organization’s Ninth Revision), as for other stroke complications such as aphasia, dysphagia, etc. This lack should be filled to start a process of improving the care of the person with spasticity.

In addition to the limited number of patients treated, a further important problem highlighted by the literature and underlined by the panel of experts is the high number of patients who discontinued BoNT-A treatment. It has been demonstrated in a pharmacoeconomic study conducted in Australia that continuing treatments beyond the fourth cycle is a cost-effective strategy [41]. Santamato and co-workers clearly showed, in a study conducted during the COVID-19 pandemic, how discontinuation of the BoNT-A treatments was associated with the worsening of perceived spasticity and associated loss of independence [39]. A recent study investigated the reasons for BoNT-A treatment discontinuation in subjects affected by spasticity post-stroke as well as other neurological conditions. Regarding stroke, subjects’ logistics reasons and clinical worsening were the most important causes of discontinuation whereas orthopedic surgeries and intrathecal baclofen therapy were frequently a reason for discontinuation for spinal cord injury and traumatic brain injury [35].

Education is crucial and affects the proper application of the current guidelines and future prospects. As indicated by the Royal College of Physicians guidelines, referral by experienced physicians and physiotherapists is recommended for patient selection, selection of appropriate pharmacological and physiotherapeutic treatment, and to decide on follow-up time [10]. The number of clinicians trained in neurological rehabilitation and specifically in the management of spasticity in general, with specific training for the injection of the botulinum toxin, is not sufficient for the number of people who may benefit from it. Moreover, specialty schools do not prepare trainees for the treatment of spasticity in a consistent manner, particularly for the clinical and practical aspects. The panel of experts suggests the involvement of scientific societies to multiply practical educational initiatives for clinicians involved in the multidisciplinary management of post-stroke spasticity.

Furthermore, it is necessary to improve and spread awareness of the need for taking charge and treatment of spasticity both among patients and caregivers and by health personnel involved in territorial care (e.g., nurses and physiotherapists).

In this case, it would be desirable to involve patients and citizen associations to raise awareness among policymakers.

The proper application of the guidelines on the management of spasticity is potentially slowed by the non-homogeneous mode of reimbursement of botulinum toxin, allowed in Italy for public neurorehabilitation hospitals, but not for private ones. Spasticity management and in particular BoNT-A use is a common issue not only in Italy: Schnitzler and colleagues calculated costs for spasticity treatment with BoNT-A; they concluded that the daily cost of BoNT-A treatment for spasticity is reasonable, but the treatment is costly for French hospitals due to the level of reimbursement by the national health insurance [48]. This can lead to a treatment discrepancy based not on medical choice but on the type of hospital or the type of setting the patient is in. An efficacy study with economic analysis is ongoing and results might provide the evidence needed for reimbursement schemes to modify funding policy for BoNT-A in post-stroke spasticity [5].

In any case, the assessment of the direct costs (e.g., adjunctive physiotherapy for contractures, greater assistance for the loss or reduction of a function) borne by the patients and the indirect costs (e.g., days of work lost by the patient and family) deriving from the failure to treat spasticity are poorly considered in clinical trials. It is noteworthy that correct spasticity management is an unmet need in community-dwelling stroke patients [49]. Kim and colleagues found that the presence of one or more unmet needs for rehabilitative management in common health-related problems (i.e., spasticity, pain, anxiety/depression, etc.) are independent negative predictors of individuals’ quality of life [50].

Recent research explored the long-term clinical and economic outcomes of post-stroke spasticity, finding that BoNT-A therapy plus rehabilitation lead to a risk reduction of 8.8% for all-cause mortality, and an increase of 59% in quality-adjusted life-years compared with rehabilitation therapy alone [42]. Lindsay et al. evaluated the cost-consequence of an early BoNT-A treatment in a stroke unit for subjects at risk of contracture and they found interesting results: contracture treatment costs were reduced [43].

The panel of experts identified the need for specific parameters to better understand disease burden, health budget impact, and direct and indirect costs of post-stroke spasticity.

## 5. Conclusions

In conclusion, the present research identified some areas that need to be implemented with the aim to improve the spasticity treatment pathway.

In particular, it is important to define the treatment path of spasticity for continuity of care (i.e., continuity of path, quality/appropriateness of delivery). There is a need to establish, strengthen, and implement the spasticity clinic: a multidisciplinary clinic involving a rehabilitation consultant, physiotherapist, an occupational therapist, and a nurse, dedicated to the evaluation and treatment of patients with spasticity.

Medical and non-medical communities need to raise awareness and educate: awareness on the correct management of patients affected by post-stroke spasticity at all levels (patients, caregivers, family physicians, and physiotherapists to the correct identification of the problem, acute facilities, neuro-rehabilitative facilities, and neurological and physiatrist clinics for the treatment of spasticity).

Furthermore, it is important to make profitable and valuable the medical expertise of spasticity treatment (e.g., ICD 9 codes, consider the opportunity to include “Spasticity” among the sequelae of cerebrovascular diseases, emphasize the importance of specialization/expertise of spasticity care in accreditation procedures).

Finally, for patients living at home and that cannot reach the clinic for medical reasons it is important to facilitate the acquisition and management of the botulinum toxin on the territory with domiciliary units for spasticity treatment.

## Figures and Tables

**Table 1:** Preliminary in-depth interviews questionnaire provided to the Expert Panel members.

Key Questions to the Expert Panel
(1) To date, what are the main critical points and barriers that limit the correct diagnosis?
(2) To date, what are the main critical points and barriers to access to treatment?
(3) In the current scenario, how is the outpatient service of botulinum toxin injection remunerated?
(4) In the light of the analysis in the above questions, how do we ensure that patients with post-stroke spasticity are diagnosed and have access to continuous (chronic) appropriate treatment?
(5) In the current scenario, what characteristics an organizational network should have to efficiently taking care of patients suffering from post-stroke spasticity (roles and responsibilities).
(6) How can the actual performance of the take-over system be measured?

**Table 2:** Summary of the selected research studies on the management of post-stroke spasticity.

Selected Papers	Title	Type of Study	OCBM Scale	Objective	Main Findings
Christofi G et al., 2018 [4]	Improving the Management of Post-Stroke Spasticity: Time for Action.	Consensus report	Level 5	To identify barriers to appropriate referral and treatment for patients with PSS and present solutions that address these in a pragmatic way.	Key barriers, throughout the patient journey prioritized by the panel, broadly related to lack of awareness and knowledge of spasticity, insufficient access to spasticity services, and a lack of standardized processes/pathways.
Bavikatte G et al., 2021 [3]	Early Identification, Intervention and Management of Post-stroke Spasticity: Expert Consensus Recommendations.	Consensus report	Level 5	The proposed system, based on clinical evidence, expert consensus, and recent clinical guidelines, provides simple and straightforward criteria for management, multidisciplinary consultation, and referral to specialist spasticity services.	The Expert Consensus, through several rules, concludes that effective and timely intervention aims to increase functional abilities, improve personal care, and impact quality of life.
Picelli A et al., 2017 [16]	The Italian real-life post-stroke spasticity survey: unmet needs in the management of spasticity with botulinum toxin type A.	Survey	Level 5	The main aim of this national survey was to provide an overview of some important issues concerning the use of BoNT-A to treat patients with PSS, and to highlight related unmet needs.	The management of PSS has several unmet needs that, were they addressed, might improve these patients’ clinical outcomes and quality of life. These needs concern patient follow-up, where a clearly defined pathway is lacking; furthermore, there is a need to use maximum doses per BoNT-A treatment and to ensure early intervention on PSS.
Francisco GE et al., 2021 [8]	A practical guide to optimizing the benefits of post-stroke spasticity interventions with botulinum toxin A: An international group consensus.	Consensus report	Level 5	This consensus paper from the international group of experts does not replicate information published elsewhere, but instead aims to provide practical advice to help optimize the use of BoNT-A and maximize clinical outcomes.	The use of BoNT-A and innovative techniques have facilitated a more individualized approach to the treatment of PSS, which provides physicians with the opportunity to optimize outcomes and address multiple goals.
Picelli A et al., 2021 [23]	Early Botulinum Toxin Type A Injection for Post-Stroke Spasticity: A Longitudinal Cohort Study.	Multicenter, longitudinal, cohort study	Level 3	The aim was of the study to determine whether the length of time between stroke onset and initial BoNT-A injection has an effect on outcomes after PSS treatment.	The study findings suggest that BoNT-A treatment for PSS should be initiated within 3 months after stroke onset in order to obtain a greater reduction in muscle tone at 1 and 3 months afterward.
Lazzaro C et al., 2020 [28]	Abobotulinum toxin A and rehabilitation vs. rehabilitation alone in post-stroke spasticity: A cost-utility analysis.	Cost-utility study	Level 3	This is the first Italian economic evaluation aimed at investigating the costs and QALYs of rehabilitation + BoNT-A (rehab/aboBoNT-A) vs. rehabilitation (rehab) in Italy, via a 2-year, model-based cost-utility analysis (CUA) in post-stroke spasticity in Italy.	Rehabilitation combined with abobotulinum toxin A is a cost-effective healthcare program for treating patients with post-stroke spasticity in Italy, for both the Italian National Health Service and society.
Rychlik R et al., 2016 [29]	Quality of life and costs of spasticity treatment in German stroke patients.	Prospective, multicenter, non-interventional parallel-group study	Level 3	To gather routine clinical practice data on post-stroke spasticity patients and their treatments in Germany. Efficacy, impact on quality of life and costs over a one-year treatment period were analyzed.	In this study, incobotulinum toxin A treatment demonstrated superior results in muscle tone reduction compared to conventional therapy and significantly improved functional impairment as well as quality of life. In the investigator’s view, the results underline the level A recommendation of national and international guidelines for the treatment of post-stroke spasticity with botulinum toxin.
Forsmark A et al., 2020 [30]	Inequalities in the pharmacologic treatment of spasticity in Sweden—health economic consequences of closing the treatment gap.	Comprehensive overview	Level 5	Sweden lacks national treatment guidelines regarding the management of spasticity, leaving room for local variations in clinical practice: a marked variation in BoNT-A treatment of adult spasticity was observed.	The results from the current study show marked regional differences regarding BoNT-A spasticity treatment in Sweden, which also apply to other pharmacological treatments. The emerging explanation of the observed variation seems to be a lack of evidence-based central guidelines, training in spasticity care, and up-to date clinical expertise.
Sandrini G et al., 2018 [31]	Management of spasticity with onabotulinumtoxinA: practical guidance based on the Italian real-life post-stroke spasticity survey.	Survey	Level 5	The aim of the paper is to provide practical guidance on the management of adult spasticity based on the unmet needs in the management of spasticity with botulinum toxin type A identified by the Italian Real-Life Post-Stroke Spasticity Survey.	All the members of the stroke care team should be aware of the early predictors of post-stroke spasticity; early predictors of spasticity should be evaluated within a few days of the onset of the stroke, and reported in the letter of discharge from the Stroke Unit. Stroke patients should be referred to spasticity services that have adequate facilities and multidisciplinary teams with the necessary training, competence, and expertise.
Demetrios M et al., 2013 [32]	Multidisciplinary rehabilitation following botulinum toxin and other focal intramuscular treatment for post-stroke spasticity.	Systematic review	Level 1	To assess the effectiveness of multidisciplinary rehabilitation, following BoNT and other focal intramuscular treatments such as phenol. To explore what settings, types, and intensities of rehabilitation programs are effective.	There was ‘low level’ evidence for the effectiveness of outpatient MD rehabilitation in improving active function and impairments following BoNT for upper limb spasticity in adults with chronic stroke. Settings, modalities, and therapy approaches are unclear.
Wissel J et al., 2022 [33]	Assessment, goal setting, and botulinum neurotoxin a therapy in the management of post-stroke spastic movement disorder: updated perspectives on best practice.	Non-systematic review	Level 5	The aim of the review is to discuss predictors, early identification, clinical assessments, goal setting, and management in a multi-professional team for early and chronic management of PS-SMD	BoNT-A to manage emerging and establishing post-stroke spastic movement disorder is recommended, safe, and dose-dependent effective local therapy. BoNT-A treatment improves activities of daily living and quality of life, especially when patient-centered goal setting in a multi-professional team and adjunctive treatment to BoNT-A is applied.
Williams G et al., 2022 [34]	A synthesis and appraisal of clinical practice guidelines, consensus statements and Cochrane systematic re-views for the management of focal spasticity in adults and children.	Systematic review	Level 2	To review the existing clinical practice guidelines, consensus statements and Cochrane systematic reviews for the PSS and to generate a single synthesized guideline	PSS management should be provided by a multi-disciplinary team; therapy should be goal-directed; PSS goals should be developed in conjunction with the patient and family; in PSS treatment follow-up evaluations are of great importance.
Cinone N et al., 2022 [35]	Reasons and Determinants of BoNT-A Treatment Discontinuation in Patients Living with Spasticity: A 10-Year Retrospective Analysis.	Retrospective Study	Level 3	to evaluate the reasons and determinants of BoNT-A discontinuation.	For stroke patients’ logistics reasons and clinical worsening were the most important causes of discontinuation.
Raluy-Callado M et al., 2018 [36]	A retrospective study to assess resource utilization and costs in patients with post-stroke spasticity in the United Kingdom.	Retrospective study	Level 3	To assess the differences in healthcare resource utilization between patients who do and do not develop PSS in the UK.	Stroke patients who develop spasticity use twice as much economic resources as patients who do not develop it, particularly for hospital readmissions.
Turner-Stokes L et al., 2018 [37]	A comprehensive person-centered approach to adult spastic paresis: a consensus-based framework.	Consensus report	Level 5	To develop a consensus-based framework towards “person-centered” medicine for the complex management of spastic paresis and to include an educative process that engages care providers and patients and encourages them to participate actively in the long-term management of spasticity.	Care focused on patient priorities. Definition of objectives, negotiation, and measurability of the same are priorities. The family’s ability to carry out self-rehabilitation must be considered and the cognitive, neuropsychological, and behavioral issues of rehabilitation must be taken into consideration.
Sunnerhagen KS et al., 2013 [38]	Enhancing patient-provider communication for long-term post-stroke spasticity management.	Non-systematic review	Level 5	To discuss patient-provider communication and its role in PSS rehabilitation within the context of patient-centered health care.	Areas to be improved: involving family members; educating patients and family members on stroke and rehabilitation and establishing a common definition for long-term goals. Increased communication among physicians, patients, and payers may bridge some of the gaps and increase the effectiveness of PSS rehabilitation and management.
Santamato A et al., 2021 [39]	Discontinuation of botulinum neurotoxin type-A treatment during COVID-19 pandemic: an Italian survey in post stroke and traumatic brain injury patients living with spasticity.	Survey	Level 5	To evaluate the impact of discontinuation of BoNT-A treatment on spasticity during the COVID-19 quarantine.	The discontinuation of BoNT-A treatment was associated with a worsening of perceived spasticity and associated loss of independence.
Jacinto J et al., 2020 [40]	Patient Perspectives on the Therapeutic Profile of Botulinum Neurotoxin Type A in Spasticity.	Survey	Level 5	To evaluate patient perceptions of the impact of spasticity and the waning of BoNT-A therapeutic effects	Symptom re-emergence is common and has a significant impact on quality of life. Greater patient/clinician awareness of this therapeutic profile should lead to a better level of overall satisfaction with treatment, informed therapeutic discussions, and treatment schedule planning.
Makino K et al., 2019 [41]	Cost Effectiveness of Long-Term Incobotulinum toxin-A Treatment in the Management of Post-stroke Spasticity of the Upper Limb from the Australian Payer Perspective.	Retrospective study	Level 3	Pharmacoeconomics study on BoNT-A treatment duration in Australia.	In Australia, BoNT-A treatment is restricted to four cycles of BoNT treatment irrespective of the subject’s response or clinical needs. This study demonstrated that in well-selected subjects more than four cycles can be cost-effective.
Fheodoroff K et al., 2022 [42]	Modelling Long-Term Outcomes and Risk of Death for Patients with Post-Stroke Spasticity Receiving Abobotulinum toxin A Treatment and Rehabilitation Therapy.	Non-systematic review	Level 5	To model the long-term clinical and economic outcomes of post-stroke spasticity.	BoNT-A plus rehabilitation therapy led to a risk reduction of 8.8% for all-cause mortality, and an increase of 13% in life-years and 59% in quality-adjusted life-years compared with rehabilitation therapy alone.
Lindsay C et al., 2023 [43]	Estimating the cost consequence of the early use of botulinum toxin in post-stroke spasticity: Secondary analysis of a randomised controlled trial.	Randomized controlled trial	Level 2	To evaluate the cost-consequence of an early BoNT-A treatment in the acute stroke unit.	An early spasticity treatment in stroke patients at risk of contractures with botulinum toxin leads to a significant reduction in contracture costs.

Table Legend: OCBM; Oxford CEBM Levels of Evidence. Level 1: systematic reviews of randomized controlled trials (RCTs). Level 2: RCTs or observational studies with a “dramatic” effect. Level 3: cohort/follow-up studies. Level 4: case-control studies, case-series studies. Level 5: mechanism-based reasoning (expert opinion). The level may be decreased based on the quality of the study, its imprecision, the inconsistency between the studies, or the modest “effect size” (low clinical relevance of the results); the level can be increased if there is an important “effect size”. A systematic review is generally superior to a single study.

**Table 3:** Summary of the selected clinical guidelines on the management of post-stroke spasticity.

Guideline	Society/Association, Year	Main Recommendations/Statements
Royal College of Physicians, 2018 [10]	Royal College of Physicians, 2018	The purpose of these guidelines is to provide clinicians with the knowledge and tools to use BoNT-A appropriately in focal spasticity. The principles for successful intervention are: - appropriate patient selection - establishment of clear goals for treatment - clear establishment of the immediate and ongoing treatment program. Local intramuscular injection of BoNT-A is an established, well-tolerated treatment in the pharmacological management of focal spasticity. There is a strong body of Level I evidence for its effectiveness in the management of both upper and lower limb spasticity. Treatment goals should be agreed upon between the team and the patient and/or their family and documented.
Winstein CJ, et al., 2016 [44]	American Heart Association (AHA), American Stroke Association (ASA), 2016	Botulinum toxin injection can be useful to reduce severe hypertonicity in hemiplegic shoulder muscles. Targeted injection of botulinum toxin into localized upper limb muscles is recommended to reduce spasticity, improve passive or active range of motion, and improve dressing, hygiene, and limb positioning. Targeted injection of botulinum toxin into lower limb muscles is recommended to reduce spasticity that interferes with gait function.
Hebert D, et al., 2015. [45]	Canadian stroke best practice recommendations	Chemodenervation using botulinum toxin can be used to reduce spasticity, increase range of motion, and improve gait, for patients with focal and/or symptomatically distressing spasticity
Smith L. 2010 [46]	SIGN Guidelines, Scotland	A Clostridium botulinum toxin type A may be considered for use to relieve spasticity following stroke where it is causing pain or interfering with physical function and the ability to maintain hand hygiene; injections may need to be repeated every three to four months and should be discontinued if lack of efficacy; botulinum toxin should only be used by those with appropriate training and care is required with the administration as the unit dosage of botulinum toxin differs between manufacturers.
Stroke Foundation of New Zealand and New Zealand Guidelines Group. 2010 [47]	Stroke Foundation of New Zealand and New Zealand Guidelines Group	Botulinum toxin A should be trialed in conjunction with rehabilitation therapy which includes setting clear goals.

## Data Availability

Not applicable.
